# Indigenous–non-Indigenous disparities in health and social outcomes 5 years after first episode psychosis: national cohort study

**DOI:** 10.1192/bjo.2024.827

**Published:** 2024-12-20

**Authors:** Ruth Cunningham, Frederieke Petrović-van der Deen, Sheree Gibb, Marie Crowe, Jenni Manuel, Suzanne Pitama, Sue Crengle, Richard Porter, Cameron Lacey

**Affiliations:** Department of Public Health, University of Otago, Wellington, New Zealand; Department of Psychological Medicine, University of Otago, Christchurch, New Zealand; Department of Māori/Indigenous Health Innovation (MIHI), University of Otago, Christchurch, New Zealand; Department of Preventive and Social Medicine, Dunedin School of Medicine, University of Otago, Dunedin, New Zealand; Specialist Mental Health Service, Te Whatu Ora, Christchurch, New Zealand

**Keywords:** Psychotic disorders/schizophrenia, social functioning, ethnic disparities, Indigenous health, big data

## Abstract

**Background:**

There are ethnic differences, including differences related to indigeneity, in the incidence of first episode psychosis (FEP) and pathways into care, but research on ethnic disparities in outcomes following FEP is limited.

**Aims:**

In this study we examined social and health outcomes following FEP diagnosis for a cohort of Māori (Indigenous people of New Zealand) and non-Māori (non-Indigenous) young people. We have focused on understanding the opportunities for better outcomes for Māori by examining the relative advantage of non-Māori with FEP.

**Method:**

Statistics New Zealand's Integrated Data Infrastructure was accessed to describe mental health and social service interactions and outcomes for a retrospective FEP cohort comprising 918 young Māori and 1275 non-Māori aged 13 to 25 at diagnosis. Logistic regression models were used to examine whether social outcomes including employment, benefit receipt, education and justice involvement in year 5 differed by indigeneity.

**Results:**

Non-Māori young people were more likely than Māori to have positive outcomes in the fifth year after FEP diagnosis, including higher levels of employment and income, and lower rates of benefit receipt and criminal justice system involvement. These patterns were seen across diagnostic groups, and for both those receiving ongoing mental healthcare and those who were not.

**Conclusions:**

Non-Māori experience relative advantage in outcomes 5 years after FEP diagnosis. Indigenous-based social disparities following FEP urgently require a response from the health, education, employment, justice and political systems to avoid perpetuating these inequities, alongside efforts to address the disadvantages faced by all young people with FEP.

Indigenous populations in New Zealand, Australia and the USA experience higher rates of psychotic illness than non-Indigenous (settler) populations in those countries, in line with evidence of a higher incidence of psychosis among other marginalised ethnic groups.^1–5^ There is also emerging evidence that adverse pathways into care and higher rates of coercive care are experienced by Indigenous people with psychosis.^6^ Experience of psychotic illness is associated with restricted life chances including low rates of employment and high rates of criminal justice system involvement.^7^ However, relatively little is known about the course of psychosis in ethnic minority groups compared with more advantaged groups,^8^ and even less is known about outcomes for Indigenous populations. Understanding differences in treatment and social outcomes for marginalised ethnic and Indigenous groups is a vital first step towards equitable outcomes of care.^9^

Māori, the Indigenous people of New Zealand, account for 16.5% of the population^10^ and are disproportionately affected by psychotic disorders,^4,11^ with young Māori being more than twice as likely to be diagnosed with FEP than non-Māori.^4^ Māori have experienced a number of injustices through colonisation, which has created and maintained economic, social and environmental disparities.^12^ The concept of non-Indigenous privilege denotes the advantage of those that do not have a history of and ongoing exposure to colonisation or the harmful effects it has on psychosis outcomes. Our previous work found non-Indigenous youth had higher rates of educational enrolment and employment and were less likely to have contact with child protection services and the criminal-justice system in the year preceding FEP diagnosis, compared with Indigenous youth.^13^ Understanding the way in which this advantage persists following FEP diagnosis and affects recovery as measured through social and functional outcomes is important for identifying opportunities to improve outcomes for Indigenous youth. In the present study, we examine social outcomes (employment, benefit receipt, criminal justice involvement) in the fifth year post diagnosis for the previously established cohort of over 2000 non-Māori and Māori youth diagnosed with FEP in New Zealand between 2009 and 2012.^4^

## Method

### Study population

We previously identified a retrospective cohort of 2412 13- to 25-year-old individuals diagnosed with FEP between 2009 and 2012.^4^ To develop this cohort we used health record data (specifically specialist mental health service use and diagnosis data) held in the Statistics New Zealand Integrated Data Infrastructure (IDI) to identify individuals with a first recorded diagnosis of psychosis between the ages of 13 and 25 during the study period. The IDI is a large database containing anonymously linked individual-level microdata about people and households in New Zealand from government and non-government agencies and surveys, including data on public health service contacts.^14^ There are eight broad categories of data: population, community, health, social services, housing, justice, income and employment, and education. Data can be linked through a central ‘spine’ that aims to capture all people who have ever been resident in New Zealand.^14^

For the analyses presented in this paper, we excluded cohort members who had spent more than a quarter of the follow-up period (the 5 years post diagnosis) abroad or who had died during this period, as we did not have complete information on their service use or outcomes following diagnosis. National border movement data were used to calculate time spent overseas. This study is reported according to the Strengthening the Reporting of Observational Studies in Epidemiology (STROBE) statement.^15^

Sociodemographic information about the cohort including age at diagnosis, gender, ethnicity and if applicable, date of death was derived from IDI's personal details table.^16^ Ethnicity information in this table is ranked according to data source quality with information from the 2013 Census ranked highest,^17^ and people can have more than one ethnic group recorded. Using this information, a dichotomous variable for Māori ethnicity was created. The New Zealand Deprivation 2013 Score (NZDep2013),^18^ an area-based measure of deprivation, was calculated for each individual's meshblock (small area) of usual residence recorded before and closest to their date of FEP diagnosis date, and grouped into the quintiles 1 (least deprived) to 5 (most deprived).

### Service patterns and outcomes 5 years post FEP diagnosis

Statistics New Zealand IDI was used to explore social and health service contacts and outcomes 5 years after FEP diagnosis for our cohort. For this study, we accessed linked health service contact, income and employment, border movement and police, justice and corrections data for our cohort from IDI's September 2018 refresh.^14^

For this paper we focused on social outcomes indicative of social inclusion and recovery in the fifth-year post FEP diagnosis. The outcomes chosen were: having received sickness or unemployment benefit for more than 6 months in year 5; having been in employment for more than 6 months in year 5; not having been in education, employment or training (NEET); and interactions with the justice system including having been proceeded against by police, charged by the Ministry of Justice or managed by the Department of Corrections. More details about these measures can be found in Supplementary Table 1 available at https://doi.org/10.1192/bjo.2024.827. The fifth year was chosen as the maximum follow-up time available in our data, in order to examine recovery outcomes beyond the period of acute management following diagnosis.

### Analyses

Outcomes were compared between non-Māori and Māori youth and presented as proportions, crude odds ratios and odds adjusted for confounding by differences in age, gender and deprivation using logistic regression models. Fewer than six individuals had missing information for area-level deprivation and were therefore excluded from these models.

Differences in the relationship between ethnicity and outcomes between diagnostic groups were further investigated using stratified analyses, with separate models for schizophrenia, bipolar disorder, substance-induced psychotic disorder, other psychosis-related diagnosis (e.g. psychotic depressive disorder, schizoaffective disorder, organic psychotic disorder and other psychotic disorder) and non-specific psychosis-related diagnosis only (where no other psychosis-related diagnoses were found), in order to investigate the possibility of confounding or effect modification by diagnosis (see Supplementary Table 2 for included ICD-10 and DSM-IV codes). Diagnosis information from the diagnosis date and the first 5 years following FEP diagnosis was used. Because individuals had multiple diagnoses recorded over this time period, a diagnosis prioritisation process was applied before grouping as above, prioritising more specific and more severe diagnoses. Diagnoses were prioritised in the following order: schizophrenia, bipolar disorder, schizoaffective disorder, psychotic depressive disorder, substance-induced psychotic disorder, other psychotic disorder, organic psychotic disorder and non-specific psychotic disorder.

The relationship between ongoing mental health service use and outcomes by ethnicity was similarly explored through stratified analysis. Health service measures indicative of ongoing receipt of acute mental healthcare were specialist mental health service contact in year 5 and mental healthcare received in an in-patient setting in year 5. A three-level variable for mental healthcare receipt was created: no specialist care received in year 5, out-patient care only received in year 5, in-patient care received in year 5 (see Supplementary Table 3 for more details about this measure). Data preparation and analyses were performed using SAS Enterprise Guide version 7.1 for Windows within Stats NZ's IDI environment.

### Ethical statement

The authors assert that all procedures contributing to this work comply with the ethical standards of the relevant national and institutional committees on human experimentation and with the Helsinki Declaration of 1975, as revised in 2013. All procedures involving human subjects/patients were approved by University of Otago Ethics Committee (HD/18/065).

### Role of the funding source

The funders had no role in the design, conduct or interpretation of the research.

## Disclaimer

These results are not official statistics. They have been created for research purposes from the IDI which is carefully managed by Stats NZ. For more information about the IDI please visit https://www.stats.govt.nz/integrated-data/. The results are based in part on tax data supplied by the Inland Revenue to Stats NZ under the Tax Administration Act 1994 for statistical purposes. Any discussion of data limitations or weaknesses is in the context of using the IDI for statistical purposes, and is not related to the data's ability to support Inland Revenue's core operational requirements.

## Relevance

This is the first study that utilises a national cohort study design to report ethnic inequities in health and social outcomes 5 years following diagnosis with first episode psychosis (FEP) and focuses on Indigenous people. Analyses showed relative non-Indigenous advantage in positive social outcomes. Non-Indigenous young people with a diagnosis of FEP were more likely to have higher levels of employment and income, and lower rates of benefit receipt and criminal justice system involvement compared with Indigenous youth. To address inequitable outcomes, increased resourcing for Indigenous youth with FEP is required as a means of extending the same level of social advantage that non-Indigenous youth have in recovery, alongside efforts to improve recovery outcomes for all young people with FEP.

## Results

### FEP cohort

Of the original FEP cohort of 2412 individuals (60% non-Māori, 40% Māori), 162 cohort members (120 non-Māori and 42 Māori) were excluded as they had spent more than a quarter of the 5-year follow-up period outside New Zealand. The excluded cohort members had spent on average more than 3 years abroad during the 5-year period. Two per cent of the cohort (51 people) died over the 5 years following FEP diagnosis (33 non-Māori, 18 Māori (1.8% versus 2.3%, *P* = 0.4157). The number of deaths were too small to perform any further analyses of mortality. The final cohort comprised 2193 individuals including 1275 non-Māori and 918 Māori.

Table 1 shows the demographic and diagnosis characteristics of the cohort members. Non-Māori cohort members were older and living in less deprived circumstances than Māori cohort members and were more likely to be diagnosed with bipolar 1 disorder and less likely to be diagnosed with schizophrenia.

**Table 1:** Demographic and clinical factors at first episode psychosis (FEP) diagnosis^a^

	Māori		Non-Māori	
	*N*	%	*N*	%
Total	918		1275	
Age at diagnosis				
13–15	84	9	105	8
16–19	387	42	444	35
20–22	249	27	414	32
23–25	195	21	315	25
Gender				
Male	591	65	789	62
Female	321	35	489	38
New Zealand Deprivation Index				
Quintile 1	48	5	186	15
Quintile 2	69	8	243	19
Quintile 3	114	12	255	20
Quintile 4	204	22	279	22
Quintile 5	474	52	312	24
FEP diagnosis group^b^				
Schizophrenia	471	51	444	35
Bipolar I disorder	132	14	336	26
Substance-induced psychotic disorder	51	6	60	5
Other psychotic disorder^c^	132	14	210	16
Non-specific psychotic disorder only	132	14	225	18

a. Population counts retrieved from Statistics New Zealand's Integrated Data Infrastructure are random rounded to base 3, meaning that the sum of individual variable levels may exceed the total count.

b. Including all diagnosis information over 5 years from FEP, prioritised in the order shown to provide one diagnosis per person where multiple diagnoses had been recorded.

c. Including psychotic depressive disorder, schizoaffective disorder, organic psychotic disorder or other psychotic disorder.

### Outcomes

Table 2 shows the mean annual income and proportion with each of the measured outcomes for non-Māori and Māori youth in the fifth year after FEP diagnosis. Non-Māori youth had a higher mean annual income (by New Zealand dollars 2700) and their odds of being in employment for more than half of the fifth-year post diagnosis were nearly 60% higher (adjusted odds ratio (aOR) 1.57, 95% CI 1.29–1.92) compared with Māori youth, after adjusting for sociodemographic differences. Conversely, non-Māori were much less likely to be on a benefit, not in employment, education or training, or involved with the criminal justice system. These differences remained after adjusting for differences in age, gender and deprivation between non-Māori and Māori with FEP. While adjusting for pre-existing levels of educational enrolment, employment, and contacts with child protection services and the criminal justice system in the year preceding FEP (in addition to adjusting for differences in age, gender and area deprivation) slightly reduced the observed ethnic differences in year 5 outcomes, strong significant associations remained. This suggests that pre-existing levels of social inequalities do not fully explain ethnic differences in functional outcomes at 5 years post FEP.

**Table 2:** Functional/social outcomes 5 years after first episode psychosis (FEP) diagnosis for non-Māori compared with Māori

Outcomes in year 5	Māori		non-Māori		Crude odds ratios (non-Māori compared with Māori)	Adjusted for gender, age and NZDep	Adjusted for age, gender, NZDep, education, employment and contact with CPS and CJS in the year preceding FEP
	Mean	s.d.	Mean	s.d.			
Average annual income (New Zealand dollars)^a^	17 674	10 823	20 429	15 264			
	*N*	%	*N*	%			
In employment for 6 or more months	228	25	516	40	1.99 (1.65 to 2.39)	1.57 (1.29 to 1.92)	1.41 (1.14 to 1.75)
Benefit receipt for 6 or more months	648	70	672	51	0.46 (0.39 to 0.55)	0.56 (0.47 to 0.68)	0.63 (0.51 to 0.77)
Not in employment, education or training	441	48	429	33	0.54 (0.46 to 0.65)	0.64 (0.54 to 0.78)	0.69 (0.56 to 0.84)
Justice involvement (police, justice and/or correction)	285	31	189	14	0.38 (0.31 to 0.47)	0.43 (0.34 to 0.53)	0.49 (0.39 to 0.63)

NZDep, New Zealand Deprivation Index; CPS, Child Protection Services; CJS, Criminal Justice System.

a. For those who had a recorded income, 90 were excluded (48 non-Māori, 42 Māori) because recorded income was zero, predominantly because of being in education or being imprisoned.

### Exploration of outcomes by diagnosis and treatment variables

For all diagnostic groups except substance-induced psychosis, the pattern was seen of higher rates of employment and lower rates of benefit receipt, and lower rates of NEET and justice system involvement among non-Māori in the fifth year after FEP diagnosis compared with Māori, although these differences were not always significant (see Table 3).

**Table 3:** Functional/social outcomes at year 5 by baseline first episode psychosis diagnosis for non-Māori compared with Māori

Functional/social outcome	Māori		Non-Māori		Crude odds ratios (95% CI) (non-Māori compared with Māori)	Adjusted^a^ odds ratios (95% CI)
	*N*/mean	%	*N*/mean	%		
Schizophrenia						
In employment for more than 6 months	81	17	126	28	1.92 (1.40 to 2.64)	1.46 (1.04 to 2.04)
Benefit receipt for more than 6 months	384	82	330	74	0.64 (0.47 to 0.88)	0.84 (0.60 to 1.17)
Not in employment, education or training	267	57	198	45	0.61 (0.47 to 0.80)	0.68 (0.52 to 0.90)
Justice involvement (police, justice and/or correction)	150	32	78	18	0.45 (0.33 to 0.62)	0.47 (0.34 to 0.65)
Bipolar disorder I						
In employment for more than 6 months	45	34	150	45	1.53 (1.00 to 2.32)	1.28 (0.82 to 1.99)
Benefit receipt for more than 6 months	81	61	129	38	0.36 (0.24 to 0.55)	0.40 (0.26 to 0.62)
Not in employment, education or training	48	36	81	24	0.52 (0.34 to 0.80)	0.64 (0.40 to 1.01)
Justice involvement (police, justice and/or correction)	36	27	39	12	0.36 (0.22 to 0.61)	0.37 (0.22 to 0.64)
Substance-induced psychosis						
In employment for more than 6 months	18	35	30	50	1.45 (0.68 to 3.09)	1.02 (0.41 to 2.53)
Benefit receipt for more than 6 months	24	47	30	50	0.91 (0.43 to 1.92)	1.03 (0.42 to 2.49)
Not in employment, education or training	15	29	18	30	1.08 (0.47 to 2.46)	1.48 (0.56 to 3.89)
Justice involvement (police, justice and/or correction)	21	41	15	25	0.45 (0.20 to 1.01)	0.45 (0.17 to 1.22)
Other psychosis diagnosis						
In employment for more than 6 months	42	32	102	49	2.11 (1.34 to 3.34)	1.95 (1.21 to 3.15)
Benefit receipt for more than 6 months	81	61	87	41	0.44 (0.28 to 0.69)	0.49 (0.31 to 0.78)
Not in employment, education or training	48	36	45	21	0.49 (0.30 to 0.79)	0.50 (0.30 to 0.83)
Justice involvement (police, justice and/or correction)	33	25	21	10	0.34 (0.19 to 0.63)	0.36 (0.19 to 0.69)
Non-specific psychosis diagnosis only						
In employment for more than 6 months	42	32	105	47	1.87 (1.19 to 2.94)	1.66 (1.01 to 2.73)
Benefit receipt for more than 6 months	75	57	99	44	0.59 (0.38 to 0.92)	0.64 (0.40 to 1.04)
Not in employment, education or training	48	36	57	25	0.57 (0.36 to 0.91)	0.66 (0.39 to 1.10)
Justice involvement (police, justice and/or correction)	45	34	33	15	0.33 (0.20 to 0.55)	0.38 (0.21 to 0.68)

a. Adjusted for age, gender and deprivation.

Nearly half (45–49%) of non-Māori with a diagnosis of bipolar disorder or an ‘other’ or non-specific psychosis diagnosis were employed for at least 6 months in year 5. The lowest proportion in employment for non-Māori was 28% in those with schizophrenia. In contrast, only 17% of Māori with a schizophrenia diagnosis were employed for at least 6 months of year 5, and in the other diagnosis groups 32–35% of Māori were employed. For the justice outcomes, non-Māori were less than half as likely to be involved with the criminal justice system in year 5 across every diagnosis group.

Table 4 shows the proportions of non-Māori and Māori youth who had contact with specialist mental health services in the fifth year after FEP diagnosis. Nearly half (44%) of non-Māori did not have any contact with services in the fifth year, compared with 36% of Māori, while only 13% of non-Māori had an in-patient admission in year 5. After adjusting for sociodemographic differences, non-Māori remained more likely to have no ongoing treatment and less likely to have an in-patient admission during year 5. Supplementary Table 4 shows these patterns of health service contact at year 5 by diagnosis. For schizophrenia, bipolar disorder and other psychosis diagnoses, non-Māori were less likely to have an in-patient admission in year 5. However, there was little difference between the proportion no longer in contact with services between non-Māori and Māori when diagnostic groups were examined separately, with the exception of those with bipolar disorder where a higher proportion of non-Māori were no longer in contact with services than Māori (47% versus 37%).

**Table 4:** Specialist mental health service contact in year 5 after first episode psychosis diagnosis

Health service type	Māori		Non-Māori		Crude odds ratios (non-Māori compared with Māori)	Adjusted odds ratios^a^ (non-Māori compared with Māori)
	*N*	%	*N*	%		
No contact	333	36	579	44	1.42 (1.19 to 1.68)	1.25 (1.04 to 1.51)
Out-patient contact	411	44	561	43	0.94 (0.80 to 1.12)	1.03 (0.86 to 1.24)
In-patient contact	183	20	168	13	0.60 (0.48 to 0.76)	0.64 (0.50 to 0.81)

a. Adjusted for age, gender and deprivation.

Table 5 shows the variation in functional outcomes by level of ongoing contact with mental health services for non-Māori compared with Māori. Those who continued to use services had lower rates of employment, higher rates of benefit use, and NEET and justice system involvement. Non-Māori were significantly more likely to be in employment and less likely to be on a benefit, NEET or in the criminal justice system at year 5 compared with Māori across every level of ongoing service contact; the differences between Māori and non-Māori outcomes were found for those with no ongoing contact with services, continuing out-patient contact and continuing in-patient contact, and persisted after adjustment of sociodemographic confounders. We conducted Wald joint hypothesis tests for the interactions between indigeneity and diagnosis/service use for all four outcomes measures at year 5. There was not much evidence to suggest that differences by ethnicity depended on the diagnostic group and/or service use.

**Table 5:** Functional and social outcomes 5 years after first episode psychosis diagnosis by ethnicity and level of ongoing service contact

Functional/social outcome	Māori		Non- Māori		Crude odds ratios (95% CI)	Adjusted^a^ odds ratios (95% CI)
	*N*	%	*N*	%		
No service contact in year 5						
In employment for more than 6 months	138	43	312	57	1.73 (1.31 to 2.28)	1.46 (1.08 to 1.96)
Benefit receipt for more than 6 months	168	53	180	33	0.45 (0.34 to 0.59)	0.54 (0.40 to 0.73)
Not in employment, education or training	99	31	102	19	0.49 (0.35 to 0.67)	0.59 (0.42 to 0.83)
Justice involvement (police, justice and/or correction)	72	23	45	8	0.30 (0.20 to 0.45)	0.32 (0.21 to 0.49)
Out-patient service contact in year 5						
In employment for more than 6 months	69	17	168	30	2.11 (1.54 to 2.90)	1.55 (1.11 to 2.18)
Benefit receipt for more than 6 months	327	80	366	65	0.49 (0.36 to 0.66)	0.59 (0.43 to 0.80)
Not in employment, education or training	216	53	219	39	0.60 (0.46 to 0.77)	0.69 (0.52 to 0.91)
Justice involvement (police, justice and/or correction)	132	32	84	15	0.38 (0.28 to 0.52)	0.44 (0.31 to 0.61)
In-patient admission in year 5						
In employment for more than 6 months	24	13	39	23	2.05 (1.16 to 3.61)	1.93 (1.05 to 3.56)
Benefit receipt for more than 6 months	156	85	126	75	0.58 (0.34 to 0.98)	0.57 (0.32 to 1.01)
Not in employment, education or training	111	61	78	46	0.56 (0.36 to 0.85)	0.60 (0.38 to 0.95)
Justice involvement (police, justice and/or correction)	81	44	57	34	0.65 (0.42 to 1.00)	0.68 (0.42 to 1.09)

a. Adjusted for age, gender and deprivation.

## Discussion

In this national cohort using linked administrative data, non-Māori youth with FEP had higher income, were more likely to be in employment, less likely to be on a benefit, NEET or involved with the justice system in the fifth year post FEP diagnosis than Māori, and these findings were consistent after adjusting for sociodemographic confounders. A similar pattern of differences was found across diagnosis groups, with the exception of substance-induced psychosis where no outcomes were significantly different between Māori and non-Māori. At 5 years after diagnosis, non-Māori had better outcomes than Māori for diagnoses with varying prognosis – for example bipolar disorder and schizophrenia. Differences between non-Māori and Māori social and functional outcomes were found for both those still in treatment and those no longer in treatment, with a similar magnitude of difference found in all treatment groups after adjusting for sociodemographic confounders.

Our overall findings of one-third of the total cohort in sustained employment, more than one-third not in education or employment and one-fifth involved with the criminal justice system after 5 years roughly corresponds to the findings of other long-term studies of social and vocational outcomes following FEP diagnosis.^19^ For example, a 2021 systematic review of employment and relationship outcomes after FEP diagnosis found that one-third of people were in employment after an average of 8 years from FEP diagnosis.^7^ Outcomes also vary by diagnosis – a 2006 systematic review found that approximately 24% of those with schizophrenia had positive long-term educational or employment outcomes (when restricted to representative studies such as this one), close to the 23% of those with a diagnosis of schizophrenia who were in employment at the end of our study.^20^ However, our study highlights the importance of examining follow-up by ethnicity as our findings clearly demonstrate that these outcomes vary by ethnicity, with non-Māori youth experiencing greater advantage than Māori.

Disparities in outcomes between ethnic groups have also been identified in other studies. For example, the UK-based AESOP-10 study found better social outcomes for White British people with FEP compared with Black Caribbean and Black African people, including lower rates of social disadvantage, isolation and time spent in unemployment over 10 years of follow-up.^8^ Hodgekins found that not being of minority ethnicity was associated with a better recovery trajectory over 12 months.^21^ Ajnakina et al^22^ found the 5-year trajectory of psychosis in people of White British ethnicity was characterised by shorter in-patient stays, lower rates of compulsory admission and less police involvement than Black African or Caribbean people, but did not find differences in social and functional outcomes. A large meta-analysis^7^ identified ethnicity as a moderator of relationship outcomes following FEP, but not employment outcomes, and noted that it was necessary to use crude ethnic categories (Black/White/Asian) which might not capture the complexity of the relationship between ethnicity and FEP outcomes. Ethnic majority groups diagnosed with a lifetime mental illness were also less likely to have severe and persistent disabilities from that illness.^23^

Indigenous people experience specific mental health inequities in the context of overall better health outcomes for non-Indigenous people. In Canada non-Indigenous people had half the rate of hospitalisation for mental disorders of First Nations people, with schizophrenia being one of the most common diagnoses after substance use disorders and mood disorders.^1^ In remote Australia the prevalence of psychosis in non-Aboriginal people is one-fifth of the prevalence among Aboriginal peoples,^24^ with less comorbid substance use disorder and less incarceration in non-Aboriginal peoples.^25^ However, there are to our knowledge no other studies which have explored employment and educational outcomes for Indigenous people following a diagnosis of psychosis.

The inequities identified in our study are likely in part because of the social patterning of opportunities, prior to FEP, flowing on to further inequities in recovery.^13^ Institutional racism, defined as differential access to the goods, services and opportunities of society by race,^26^ is normative, sometimes legalised and often manifests as inherited disadvantage, such as is seen in the high rates of psychosis experienced by Māori.^4^ It is structural, having been codified in our institutions of custom, practice and law. It can also manifest itself as benign inaction in the face of need. Addressing these inequities will therefore require a response that goes beyond the health system, encompassing the education, employment, justice and political systems.

The health system also has an important role to play. The disparities found in social and functional outcomes after a psychosis diagnosis may reflect the appropriateness of treatment options provided. Many studies of treatments for psychosis do not include Indigenous people, and most clinical trials generate findings that are not generalisable across ethnicity.^27^ There is also some evidence that poorer functional outcomes in FEP are associated with untreated depression.^28^ It has previously been noted that Māori have lower rates of diagnosis of comorbid affective disorders in FEP which could indicate under-appreciation of affective symptoms in Māori youth with psychosis.^28,29^

The way that care is provided affects outcomes. Recent work from the UK found that mental health services are experienced as disempowering by many Black Caribbean people receiving care for psychosis, compounding the broader social and economic disempowerment experience, and leading to a cycle of mistrust and alienation from services.^30^ Research with Māori with bipolar disorder has similarly found the delivery and scope (as well as the accessibility) of mental health services acted as a barrier to equitable care for Māori.^31^

### Strengths and limitations

This study has a number of strengths. It is a population-based sample, with near complete follow-up due to the use of administrative data. The large sample size made it possible to explore the role of a number of potential confounders and effect modifiers in the relationship between indigeneity and social outcomes 5 years after diagnosis with FEP.

This study was limited by the availability and quality of the routine administrative data used. It was not possible to explore access to different treatment types (in particular, early psychosis intervention services) by ethnicity and the impacts of treatment types on outcomes after FEP diagnosis, because specific information on the type of mental health service provided was not available. There was also limited clinical information available (with the exception of diagnoses and service use intensity) to establish whether differences in presentation or severity of psychosis between Māori and non-Māori were a factor in the differences found. It was not possible to adjust for duration of untreated psychosis which affects treatment outcomes and may be different by ethnicity. Severity measures (such as clinical rated scales) were not available in the routine data, and so differences in severity of psychotic illness as an explanation for differences between Māori and non-Māori could not be directly examined. However, diagnosis provides a proxy for severity, and it is notable that with the exception of substance-induced psychosis, a similar pattern was found across diagnostic groups, and differences were not explained by higher rates of schizophrenia diagnosis among Māori than non-Māori. Differences were also not explained by higher rates of ongoing treatment for Māori, another proxy for severity. Follow-up time was limited to 5 years because of data availability and consistency issues. Some research suggests better outcomes in terms of social functioning and employment after longer periods of follow-up. For example, recent research showed that 13 years post diagnosis close to half of people were in employment.^32^

Finally, the social outcomes were only examined in the fifth-year post FEP diagnosis, and differences in these outcomes between years 1 and 4 were not investigated. Focusing on outcomes at year 5 is a limitation of this study as it does not account for the pathways and intermediate outcomes in the first 4 years after diagnosis and the ways in which these may differ between groups.. Longitudinal analyses would capture outcomes at intermediate points and enable exploration of variations in how outcomes evolve over time. This would provide a fuller understanding of the recovery process, and the factors influencing differences in outcomes. However, outcomes at year 5 represent the results of treatment and social supports over the 5 years after diagnosis and therefore to some extent reflect the impact of trajectories over this period, and there is no evidence to suggest that longer or shorter follow-up would reduce the ethnic differences found. Further research should explore these pathways in a more detailed, longitudinal manner, examining treatment trajectories and longer-term outcomes.

This study focused on outcomes for Indigenous Māori. As reported previously, the non-Indigenous cohort were primarily of European ethnicity (80%) while 13% were Pacific and a similar proportion (11%) were in one of the Asian ethnic groups, including people reporting more than one ethnicity.^4^ Among the Māori cohort, the most common second ethnic identity was European (47%), followed by Pacific (11%). It is a limitation of this study that the non-Indigenous group included young people from marginalised groups, and separately examining outcomes for other marginalised young people might further highlight discrepancies in outcomes and more clearly demonstrate the impacts of marginalisation. However, this study did not have the statistical power to disaggregate the non-Indigenous group to examine outcomes for other marginalised groups separately. Further research exploring the experience of other marginalised groups in New Zealand including Pacific young people would provide a more nuanced understanding of social disparities and add to our understanding of the social patterning of opportunities for young people with psychosis and opportunities to improve outcomes.

### Implications for practice

This study highlights inequities for Māori in the long-term treatment outcomes of FEP. To address this, the social, educational, justice and mental health services need to address the inherent institutional racism embedded in these service structures, policies and approaches. This study shows that systemic and structural injustice apparent before FEP continues to pervade and deleteriously affect Māori outcomes 5 years after FEP. Increased resourcing for Indigenous youth with FEP, as a means of extending the same level of social advantage that non-Indigenous youth have in FEP recovery, should be investigated to see if this improves Indigenous FEP outcomes. This would require a restructuring of how services are funded and delivered. It could include concerted efforts to recruit Māori into the early psychosis workforce, lower case load numbers for those working with Indigenous youth with FEP and an increased provision of social and vocational interventions for Māori. Efforts to target the social-environmental context could also be extended to the families of Indigenous youth with FEP. Across all these systems there needs to be a coordinated approach to identify and address systemic, structural social risk factors.^33^ Concerted efforts to share power with Māori through genuine partnership in all levels of service delivery may go some way to addressing the identified disparities in outcomes following first episode psychosis.

## Supporting information

Cunningham et al. supplementary material Cunningham et al. supplementary material

## Data Availability

Access to the data used in this study was provided by Statistics New Zealand (Stats NZ) under conditions designed to give effect to the security and confidentiality provisions of the Statistics Act 1975. The results presented in this study are the work of the authors, not Stats NZ. Data are held securely within the Integrated Data Infrastructure (IDI) managed by Stats NZ. Access to the IDI is restricted to approved researchers only. Please contact the authors for further details on accessing the study data and code.
