# Indigenous–non-Indigenous disparities in health and social outcomes 5 years after first episode psychosis: national cohort study – CORRIGENDUM

**DOI:** 10.1192/bjo.2025.10064

**Published:** 2025-07-16

**Authors:** Ruth Cunningham, Frederieke Petrović-van der Deen, Sheree Gibb, Marie Crowe, Jenni Manuel, Suzanne Pitama, Sue Crengle, Richard Porter, Cameron Lacey

In the original publication of this article, the below statement cited an incorrect reference:

“In remote Australia the prevalence of psychosis in non-Aboriginal people is one-fifth of the prevalence among Aboriginal peoples”

This should have cited reference 5: Gynther B, Charlson F, Obrecht K, Waller M, Santomauro D, Whiteford H, et al. The epidemiology of psychosis in Indigenous populations in Cape York and the Torres Strait. EClinicalMedicine 2019; 10: 68–77.

The original article has been updated to reflect this and the references have been re-numbered.
