# Supplementary material for: Indigenous–non-Indigenous disparities in health and social outcomes 5 years after first episode psychosis: national cohort study
Source: BJPsych Open. 2024 Dec 20;11(1):e9. doi: 10.1192/bjo.2024.827 (PMC11733454; doi:10.1192/bjo.2024.827)
Supplement: Cunningham et al. supplementary material [file S2056472424008275sup001.docx]

**Supplementary Table 1 Social outcomes indicative of social inclusion and recovery in the fifth-year post FEP diagnosis**

| **Measure** | **Data set(s)** | **Description** |
| --- | --- | --- |
| **Employment** | Income and tax year summary data | Being employed was defined as having received income through salaries and wages, paid parental leave, or sole trading for six or more months in the fifth-year post FEP. |
| **Benefit receipt** | Income and tax year summary data | Having received tier 1 main benefit payments which are intended to meet the general costs of living (for example Jobseeker support, sole parent support, or a supported living payment) for six or more months in the fifth-year post FEP. |
| **Justice involvement** | Recorded crime offenders data | Any type of recorded alleged offence that resulted in being proceeded against by police in the fifth-year post FEP. AND/OR |
|  | Court charges data | Any type of charge processed by Criminal Courts that had been finalised with a final charge outcome in the fifth-year post FEP. AND/OR |
|  | Sentencing and remand data | Any type of management by Corrections including *community management* (home detention sentence, post detention conditions, intensive supervision, community detention, supervision, community work, periodic detention, community programme, community service, and other community), *imprisonment* (prison sentence and remanded in custody)  or *released prisoner management* (extended supervision order, released to home detention, paroled, release on conditions, and returning offender order) in the fifth-year post FEP. |
| **Not in education, employment or training (NEET)** | Primary and secondary schools data, tertiary education data, targeted training data, industry training education data and income and tax year summary data | Not having a record of being enrolled in secondary school, tertiary education, targeted training, or industry training, and no record of employment (see above measure) in the fifth-year post FEP. |

**Supplementary table 2 Included ICD-10 and DSM-IV codes for psychosis-related diagnosis (used in stratified analyses)**

| **Diagnosis group** | **Diagnosis** | **ICD-10 codes** | **DSM-V codes** |
| --- | --- | --- | --- |
| Schizophrenia | Schizophrenia | F20 | 29510-29560  29590 |
| Bipolar I disorder | Bipolar I disorder | F301-F309  F311-F312  F315-F316  F319 | 29600-29604,  29641-29644,  29654,  29660-29664,  2967-29680 |
| Substance-induced psychosis | Substance-induced psychotic disorder | F105, F115, F125, F135, F145, F155,  F165, F175, F185, F195 | 2913-2915,  29211-29212 |
| Other psychosis diagnosis | Schizoaffective disorder | F25 | 29570 |
|  | Depressive episode with psychotic symptoms | F323, F333 | 29624, 29634 |
|  | Other psychotic disorder | F22-F24, F28 | 2988, 2971,  2973 |
|  | Organic psychotic disorder | F060, F062 | 29381,29382 |
| Non-specific psychosis diagnosis only | Non-specific psychotic disorder | F29 | 2989 |

**Supplementary table 3 Measure of contact with mental health services in the fifth-year post FEP (used in stratified analyses)**

| **Type of mental health services contact** | **Data set** | **Measure** | **Excluded** |
| --- | --- | --- | --- |
| Outpatient contact only | Programme  for the Integration of Mental Health Data (PRIMHD) | Any record of contact with mental health services that was provided face-to-face or via television, video-conference link, other social media and/or e-therapy, or phone, and provided in an outpatient setting, and no record of acute inpatient care (see below) in the fifth-year post FEP. | -Recorded contacts that were SMS text message and/or written  -Recorded contacts where consumer did not attend  -Recorded contacts about consumer with consumer not being present (eg, care liaison contacts, needs assessment, court liaison attendances, support contacts for family only)  -Recorded contacts that were not submitted to PRMHD by a District Health Board (DHB). |
| Acute inpatient contact | PRIMHD | Any record of acute inpatient care in District Health Board (DHB) hospitals in the fifth-year post FEP. | -Non-DHB bed nights |
| No contact | PRIMHD | No record of in- or outpatient contact (see above) with mental health services in the fifth-year post FEP |  |

**Supplementary table 4 Health service contact at year 5 by diagnosis**

| **Health service contact at year 5** | **Māori** |  | **Non-Māori** |  |
| --- | --- | --- | --- | --- |
| **Diagnosis** | N | % | N | % |
| **Schizophrenia** |  |  |  |  |
| No contact | 90 | 19.1 | 84 | 18.9 |
| Outpatient contact only | 261 | 55.4 | 267 | 60.1 |
| Inpatient contact | 120 | 25.5 | 93 | 20.9 |
| **Bipolar I Disorder** |  |  |  |  |
| No contact | 48 | 37.2 | 159 | 47.3 |
| Outpatient contact only | 51 | 39.5 | 135 | 40.2 |
| Inpatient contact | 30 | 23.3 | 42 | 12.5 |
| **Substance-induced psychosis diagnosis** |  |  |  |  |
| No contact | 33 | 73.3 | 39 | 68.4 |
| Outpatient contact only | 12 | 26.7 | 18 | 31.6 |
| Inpatient contact | s |  | s |  |
| **Other psychosis diagnosis** |  |  |  |  |
| No contact | 69 | 52.3 | 126 | 60.0 |
| Outpatient contact only | 45 | 34.1 | 72 | 34.3 |
| Inpatient contact | 18 | 13.6 | 12 | 5.7 |
| **Non-Specific psychosis diagnosis only** |  |  |  |  |
| No contact | 78 | 60.5 | 141 | 61.8 |
| Outpatient contact only | 42 | 32.6 | 66 | 28.9 |
| Inpatient contact | 9 | 7.0 | 21 | 9.2 |
